# A nomogram for predicting overall survival in oral squamous cell carcinoma: a SEER database and external validation study

**DOI:** 10.3389/fonc.2025.1557459

**Published:** 2025-03-17

**Authors:** Ziye Xu, Manbin Xu, Zhichen Sun, Qin Feng, Shaowei Xu, Hanwei Peng

**Affiliations:** ^1^ Department of Head and Neck Surgery, Cancer Hospital of Shantou University Medical College, Shantou, Guangdong, China; ^2^ Otolaryngology Department of The First Affiliated Hospital of Shantou University Medical College, Shantou, Guangdong, China

**Keywords:** oral squamous cell carcinoma, oral cancer, overall survival, nomogram, prognosis

## Abstract

**Purpose:**

Oral squamous cell carcinoma (OSCC) often presents with unsatisfactory survival outcomes, especially in advanced stages. This study aimed to develop and validate a nomogram incorporating demographic, clinicopathologic, and treatment-related factors to improve the prediction of overall survival (OS) in OSCC patients.

**Methods:**

Data from 15,204 OSCC patients in a US database were retrospectively utilized to construct a prognostic model and generate a nomogram. External validation was performed using an independent cohort of 359 patients from a specialized cancer center in China. Prognostic factors were identified using Cox regression analysis and incorporated into the nomogram. Model performance was evaluated by concordance index (C-index), time-dependent area under the receiver operating characteristic curves (AUC), calibration plots, and decision curve analysis (DCA). A risk stratification system was developed to classify patients into high- and low-risk groups.

**Results:**

Age, sex, primary tumor site, T and N staging, and treatment modalities (including surgery, chemotherapy, and radiotherapy) were found to be independent prognostic factors. The nomogram achieved a C-index of 0.727 in the training set and 0.6845 in the validation set, outperforming the conventional TNM staging system. The nomogram’s superior predictive accuracy was confirmed by higher AUC values, better calibration, and improved clinical utility as demonstrated by DCA. Risk stratification, based on the nomogram, distinguished patients into distinct prognostic groups with significant OS differences.

**Conclusions:**

This nomogram provides an effective, personalized tool for predicting OS in OSCC. It offers clinicians a valuable aid for treatment decision-making and improves patient management.

## 1 Introduction

Oral cancer refers to a tumor that occurs on the mucosal lip, buccal mucosa, upper and lower alveolar ridge, tongue, floor of the mouth, hard palate, and other parts of the mouth, which is the most common subtype in head and neck cancer. The Global Cancer Observatory (GLOBOCAN) reported that there were 389,846 new cases and 188,438 deaths of oral cancer worldwide in 2022 (1). It is predicted that there will be 36,620 new cases and 7,930 deaths of oral cancer in the United States (2). Approximately 90% of oral malignancies are squamous cell carcinomas [oral squamous cell carcinoma (OSCC)] (3). The incidence and mortality rates of OSCC vary significantly across countries, areas, and races (4). Despite the improvement in the treatment of OSCC, including the surgical technique, chemotherapy, and radiotherapy regimens, the prognosis of OSCC remains unsatisfactory, with a 5-year overall survival (OS) of 70%–80% in the early stage and 35%–45% in the advanced stage (5). Accurate identification of high-risk patients enables surgeons to formulate more rational treatment decisions and enhances preoperative communication between medical professionals and patients along with their families. However, to date, accurate prediction of the prognosis of oral cancer remains a significant clinical challenge. Therefore, it is vital to develop an intuitive instrument that can help clinicians predict the survival of patients. The 8th Edition of the American Joint Committee on Cancer (AJCC) Staging Manual, Head and Neck Section, is recommended for assessing the prognosis of OSCC patients (6). However, the outcomes of OSCC patients are influenced by many factors, such as age, sex, race, tumor site, radiation, chemotherapy, and surgery (7, 8). Therefore, a combination of relevant clinicopathologic and therapeutic factors can provide a more reliable prediction than the single AJCC staging manual.

A nomogram derived from independent prognostic factors identified through multivariate Cox proportional hazards regression serves as a valuable tool for surgeons to estimate OS in OSCC patients. Numerous nomograms have been established to assist surgeons in prognostic evaluation across various malignancies, including breast cancer (9), gastric cancer (10), and esophageal cancer (11). Although Wang F et al. (12) previously published a nomogram for predicting OS of OSCC patients, their model lacked external validation using independent datasets, thereby limiting its generalizability to broader populations. Similarly, the predictive model proposed by Zhang X et al. (13) was constructed using single-center data, which may introduce selection bias and compromise its clinical applicability. While Wang W et al. (14) developed a model using data from Hong Kong and Australia; the limited scope of variables and suboptimal validation performance underscore the need for further refinement. Consequently, there is a critical need for a robust predictive model that incorporates a comprehensive set of variables and leverages multicenter data to enhance its accuracy and generalizability.

Since 1973, the Surveillance, Epidemiology, and End Results (SEER) database, established by the National Cancer Institute (NCI), has systematically collected and disseminated cancer incidence and survival data, encompassing approximately 48% of the US population-based cancer registries; it has become an invaluable resource widely utilized in cancer research (15). This comprehensive database has been widely used in cancer research. In this study, the SEER database, with its extensive collection of clinical cases, serves as a critical foundation for data analysis and model development.

This retrospective study aims to identify the prognostic factors for the OS of OSCC patients and to develop a nomogram by investigating the relationships between demographic features, clinicopathologic features, therapeutic information, and survival using data collected from the SEER database and the Cancer Hospital of Shantou University Medical College (CHSUMC).

## 2 Materials and methods

### 2.1 Data collection

In this study, data were extracted from the SEER database from 17 registries using SEER*Stat V8.4.3 (www.seer.cancer.gov/seerstat). Patients diagnosed with OSCC between 2010 and 2017 were included based on the following criteria: 1) diagnosis with OSCC confirmed by histopathology, 2) OSCC as the only primary malignancy, 3) cases with valid survival status and follow-up time, and 4) a minimum follow-up period of 1 month from the initial diagnosis. Meanwhile, patients were excluded if 1) they had missing information on tumor grade and/or TNM stage and 2) they had the presence of distant metastasis at the time of initial diagnosis. The SEER dataset served as the training cohort for developing a survival prediction model and constructing the nomogram.

For external validation, data from OSCC patients diagnosed between 2010 and 2020 at CHSUMC were utilized. The same inclusion and exclusion criteria were applied, with the additional exclusion of patients who had received treatment at other institutions. The last follow-up date was May 2024. Key variables analyzed included sex, age at diagnosis, race, primary site, TNM staging, histological grade, surgical information, chemotherapy, radiotherapy, survival status, and follow-up duration. The primary endpoint of this study was OS, defined as the time interval between the date of diagnosis and the date of death from any cause.

### 2.2 Data analysis

All the statistical analyses were run in RStudio V4.3.1 (https://posit.co/downloads/). *p* < 0.05 was considered statistically significant. The “X-tile” software, a bioinformatics tool designed for biomarker assessment and outcome-based cut-point optimization (16), was employed to determine optimal cutoff values by evaluating all possible partitioning methods. The R package “Compare Groups” was used to compare differences in demographic characteristics, clinical features, and treatment between the training set and validation set. The Kaplan–Meier curves (long-rank test) were plotted to compare the OS between two groups using the R packages “survival”, “readxl”, and “survminer”.

### 2.3 Cox regression and nomogram establishment

Schoenfeld residuals were used to test the proportional hazards (PH) assumption. Univariate and multivariate Cox regression analyses were run using the R package “rms” and “survival”. Variables with *p* < 0.05 in the univariate Cox regression were included in the multivariate Cox regression. Independent risk factors significantly related to the OS of OSCC patients were selected to develop a Cox regression model. Finally, the nomogram reflected that the model was plotted to predict 1-, 3-, and 5-year OS for OSCC patients using the R package “autoReg”. Another Cox regression model based on the TNM staging was also established to compare with the new model.

### 2.4 Validation

To evaluate the accuracy and effectiveness of the model, internal and external validation were both taken. The concordance index (C-index), area under the receiver operating characteristic (ROC) curve (AUC), and calibration curves were utilized to assess the reliability and accuracy of the nomogram. The net benefits of the prediction model were calculated and compared with those of the TNM staging model by plotting the decision curve analysis (DCA). Finally, the predicted points of all cases were calculated using the model and divided into the high-risk group and low-risk group via the X-tile. The OS between the two risk categories was compared using the Kaplan–Meier analysis.

## 3 Result

### 3.1 Patient characteristics

**Figure 1:**
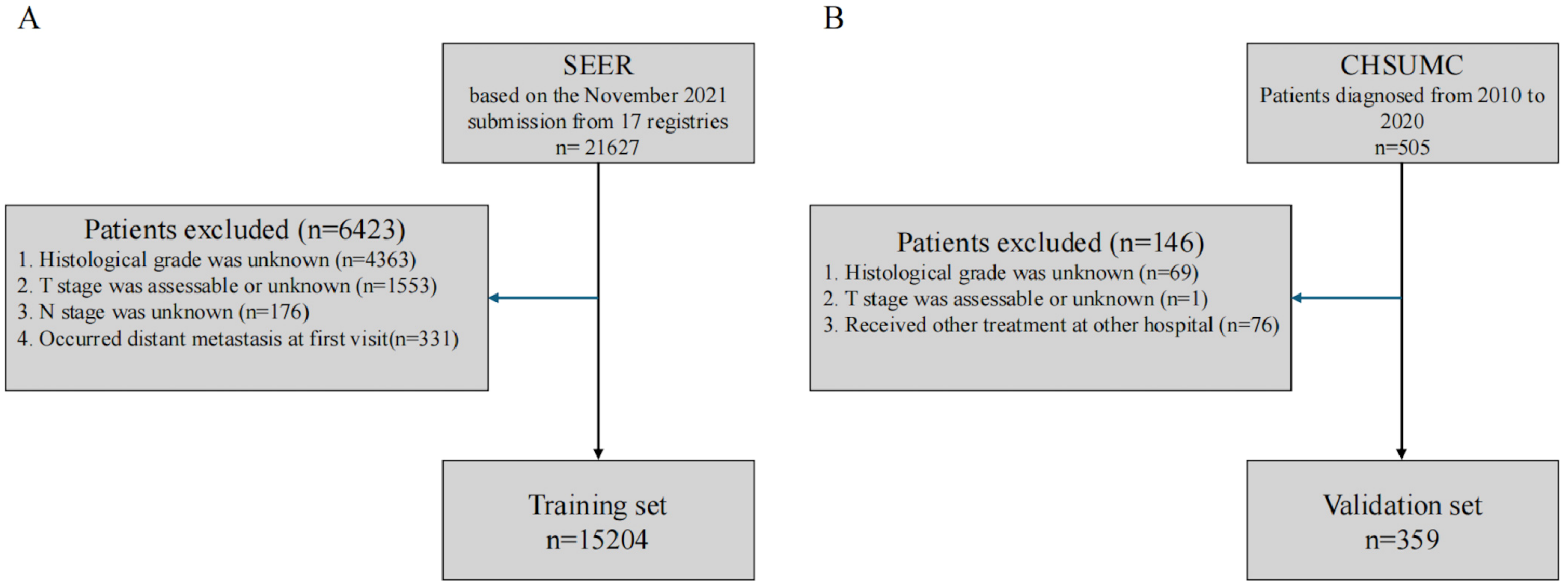
Flowchart of participant selection. **(A)** SEER database. **(B)** Cancer Hospital of Shantou University Medical College. SEER, Surveillance, Epidemiology, and End Results.

The flowchart illustrating the participant selection process is presented in **Figure 1**. A total of 15,204 patients from the SEER database (2010–2021) and 359 patients from the CHSUMC (2010–2020) were included in this study. The demographic and clinical characteristics of the patients are summarized in **Table 1**.

**Table 1:** Demographic characteristics, clinical features, and treatment information of the training set and validation set.

	SEER (training)	CHSUMC (validation)	*p*
	N = 15,204	N = 359	
Age			<0.001
<65 years	8,782 (57.8%)	226 (63.0%)	
65–79 years	4,673 (30.7%)	116 (32.3%)	
80+ years	1,749 (11.5%)	17 (4.74%)	
Sex			0.003
Female	4,904 (32.3%)	143 (39.8%)	
Male	10,300 (67.7%)	216 (60.2%)	
Race			
American Indian/ Alaska Native	107 (0.70%)	0 (0.00%)	
Asian or Pacific Islander	1,130 (7.43%)	359 (100%)	
Black	943 (6.20%)	0 (0.00%)	
White	13,024 (85.7%)	0 (0.00%)	
Site			<0.001
Floor of mouth	1,244 (8.18%)	36 (10.0%)	
Gum and other parts of the mouth	3,159 (20.8%)	105 (29.2%)	
Lip	1,252 (8.23%)	14 (3.90%)	
Tongue	9,549 (62.8%)	204 (56.8%)	
Grade			<0.001
Grade I	3,151 (20.7%)	249 (69.4%)	
Grade II	7,835 (51.5%)	89 (24.8%)	
Grade III	4,137 (27.2%)	21 (5.85%)	
Grade IV	81 (0.53%)	0 (0.00%)	
T			<0.001
T1	5,784 (38.0%)	63 (17.5%)	
T2	4,346 (28.6%)	160 (44.6%)	
T3	1,932 (12.7%)	74 (20.6%)	
T4	3,142 (20.7%)	62 (17.3%)	
N			<0.001
N0	7,643 (50.3%)	238 (66.3%)	
N1	2,091 (13.8%)	41 (11.4%)	
N2	5,175 (34.0%)	61 (17.0%)	
N3	295 (1.94%)	19 (5.29%)	
**M** M0	15,204 (100%)	359 (100%)	
Surgery			<0.001
No	4,601 (30.3%)	13 (3.62%)	
Yes	10,603 (69.7%)	346 (96.4%)	
Radiotherapy			<0.001
No	6,661 (43.8%)	283 (78.8%)	
Yes	8,543 (56.2%)	76 (21.2%)	
Chemotherapy			<0.001
No	9,605 (63.2%)	272 (75.8%)	
Yes	5,599 (36.8%)	87 (24.2%)	
**Survival months**, M(P_25_, P_75_)	48 [18;76]	58 [26,100]	<0.001

SEER, Surveillance, Epidemiology, and End Results; CHSUMC, Cancer Hospital of Shantou University Medical College.

The majority of patients in both cohorts were younger than 65 years. Specifically, 57.8% of patients in the SEER cohort and 63.0% in the CHSUMC cohort were younger than 65 years. The SEER cohort had a significantly higher proportion of patients aged 80 years and older (11.5%), compared to the CHSUMC cohort (4.74%). The difference in age distribution between the two cohorts was statistically significant (*p* < 0.001). Male patients predominated in both cohorts. In the SEER cohort, 67.7% were male, while in the CHSUMC cohort, male patients comprised 60.2% of the patients (*p* = 0.003).

In the SEER cohort, the majority of patients were White (85.7%), followed by Asian or Pacific Islander (7.43%), Black (6.20%), and American Indian/Alaska Native (0.70%). In contrast, all patients in the CHSUMC cohort were Asian, reflecting the demographic composition of the hospital’s patient population.

The tongue was the most common primary tumor site in both cohorts, accounting for 62.8% of cases in the SEER cohort and 56.8% in the CHSUMC cohort (*p* < 0.001). The CHSUMC cohort had a higher proportion of tumors located in the gum and other parts of the mouth (29.2% *vs.* 20.8%) and a slightly higher proportion of floor of the mouth (FOM) cancers (10.0% *vs.* 8.18%). Lip cancers were less common in the CHSUMC cohort (3.90%) compared to the SEER cohort (8.23%).

There was a significant difference in tumor differentiation between the two cohorts (*p* < 0.001). The CHSUMC cohort had a higher proportion of well-differentiated tumors (Grade I) at 69.4%, compared to 20.7% in the SEER cohort. Moderately differentiated tumors (Grade II) were more common in the SEER cohort (51.5% *vs.* 24.8%). Poorly differentiated tumors (Grade III and IV) were less prevalent in the CHSUMC cohort (5.85% *vs*. 27.7%).

The difference in T-stage and N-stage distributions between the two cohorts was significant (*p* < 0.001). Patients in the SEER cohort were more likely to have T1 tumors (38.0%) compared to the CHSUMC cohort (17.5%). The CHSUMC cohort had a higher proportion of T2 tumors (44.6% *vs.* 28.6%). The proportions of T3 and T4 tumors were relatively similar between the two cohorts.

A higher proportion of patients in the CHSUMC cohort were N0 (66.3%) compared to the SEER cohort (50.3%). The SEER cohort had a higher percentage of N2 patients (34.0% *vs.* 17.0%), while the N3 stage was more prevalent in the CHSUMC cohort (5.29% *vs.* 1.94%).

There were significant differences in treatment approaches between the two cohorts (*p* < 0.001). In the CHSUMC cohort, a vast majority of patients underwent surgery (96.4%) compared to 69.7% in the SEER cohort. The SEER cohort had higher rates of patients receiving radiotherapy (56.2% *vs.* 21.2%) and chemotherapy (36.8% *vs.* 24.2%) compared to the CHSUMC cohort.

The median OS for patients in the SEER cohort was 48 months, with the first quartile (Q1) and third quartile (Q3) being 18 and 76 months, respectively. In contrast, the median OS for patients in the CHSUMC cohort was 58 months, with Q1 and Q3 of 26 and 100 months, respectively.

**Figure 2:**
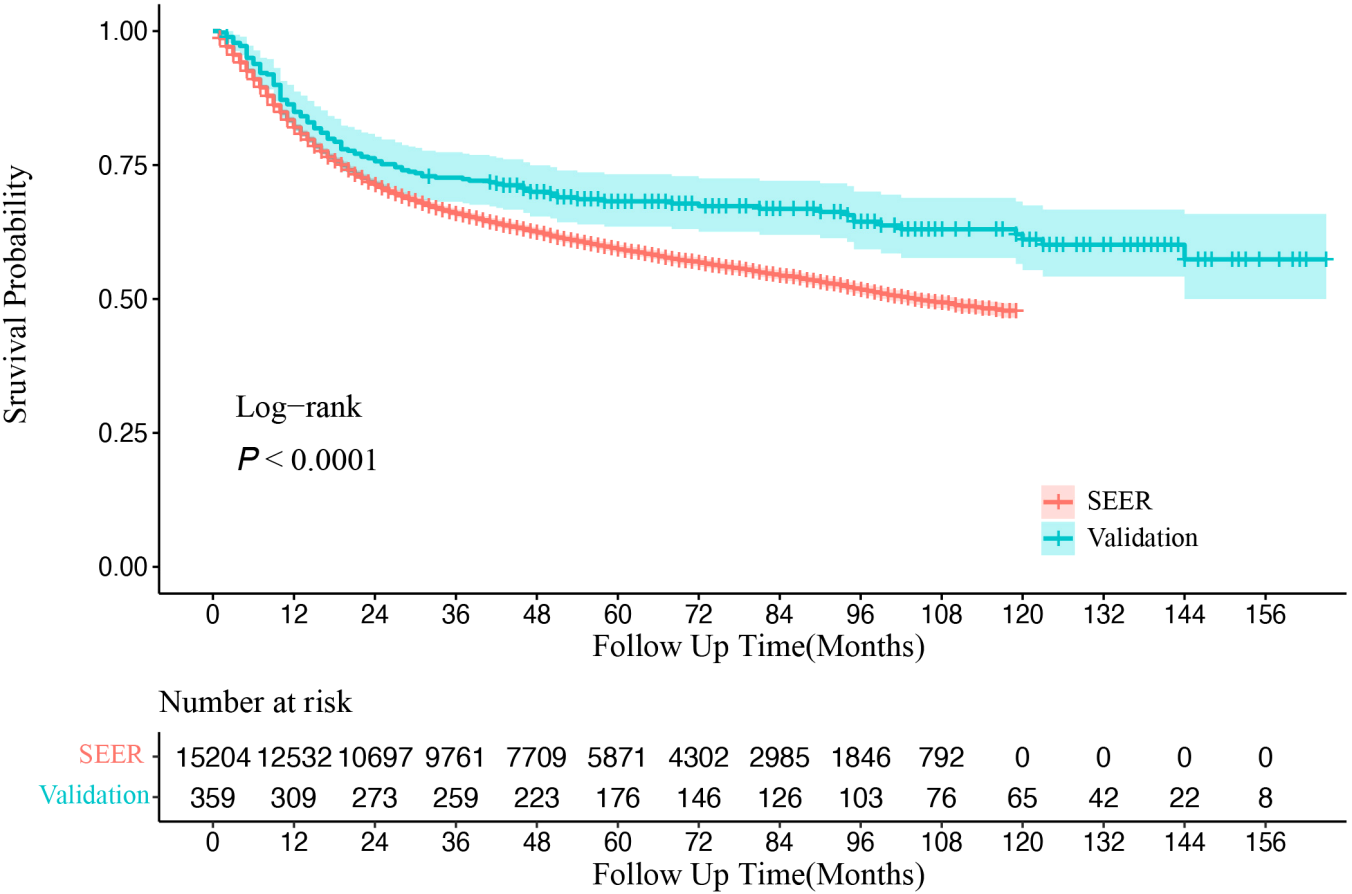
Kaplan–Meier survival curve of overall survival in SEER and CHSUMC. SEER, Surveillance, Epidemiology, and End Results; CHSUMC, Cancer Hospital of Shantou University Medical College.

The Kaplan–Meier survival analysis (**Figure 2**) revealed that OS for OSCC patients was significantly higher in the CHSUMC cohort than in the SEER cohort, with a 5-year OS rate of approximately 70% in the CHSUMC cohort and 60% in the SEER cohort (*p* < 0.0001).

### 3.2 Univariate and multivariate analyses

**Figure 3:**
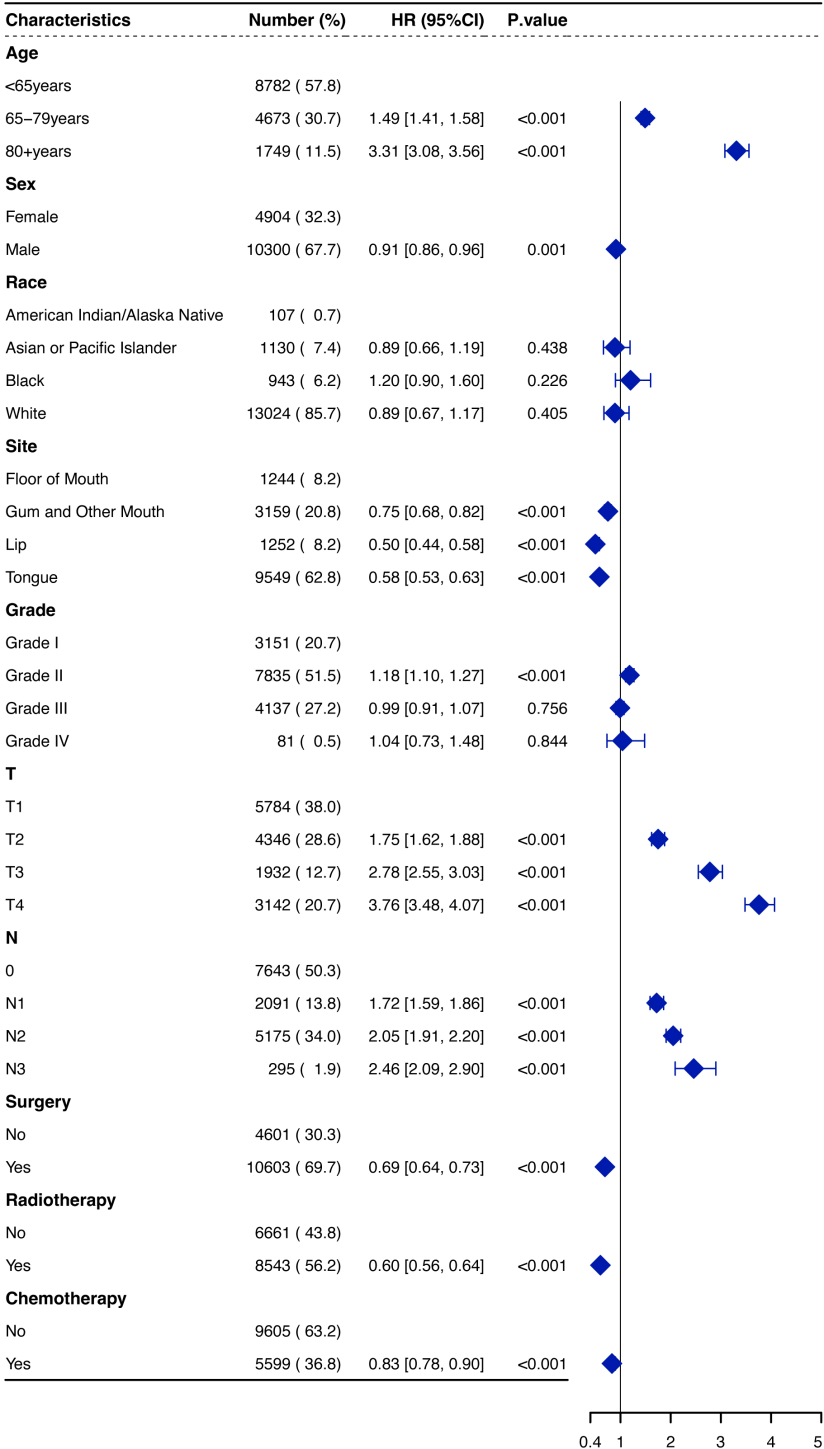
Forest plot of hazard ratios (HRs) for overall survival of OSCCpatients in the SEER database. OSCC, oral squamous cell carcinoma; SEER, Surveillance, Epidemiology, and End Results.

We assessed the PH assumption using Schoenfeld residuals and identified that the assumption was violated in certain variables, such as surgery and radiotherapy. Consequently, the hazard ratios (HRs) were interpreted as weighted averages of the time-varying HRs over the entire follow-up period (17). Both univariate and multivariate Cox regression analyses were performed to identify significant factors associated with OS in OSCC patients. Age, sex, primary site, T staging, N staging, and treatment modalities were found to be independent predictors of OS in the multivariate analysis (**Figure 3**). Increasing age was associated with a worse prognosis; patients aged over 80 years had an HR of 3.31 (95% CI: 3.08–3.56) compared to those under 65 years (*p* < 0.001). Male patients demonstrated a slightly better prognosis than female patients (HR, 0.91, 95% CI: 0.86–0.96, *p* = 0.001). The primary tumor site significantly affected survival outcomes, with lip cancers exhibiting the best prognosis and FOM cancers the poorest (HR, 0.50 for lip *vs.* FOM, *p* < 0.001). Additionally, surgical treatment emerged as a strong protective factor (HR, 0.69, 95% CI: 0.64–0.73, *p* < 0.001), underscoring its substantial impact on improving patient survival.

### 3.3 Nomogram establishment

**Figure 4:**
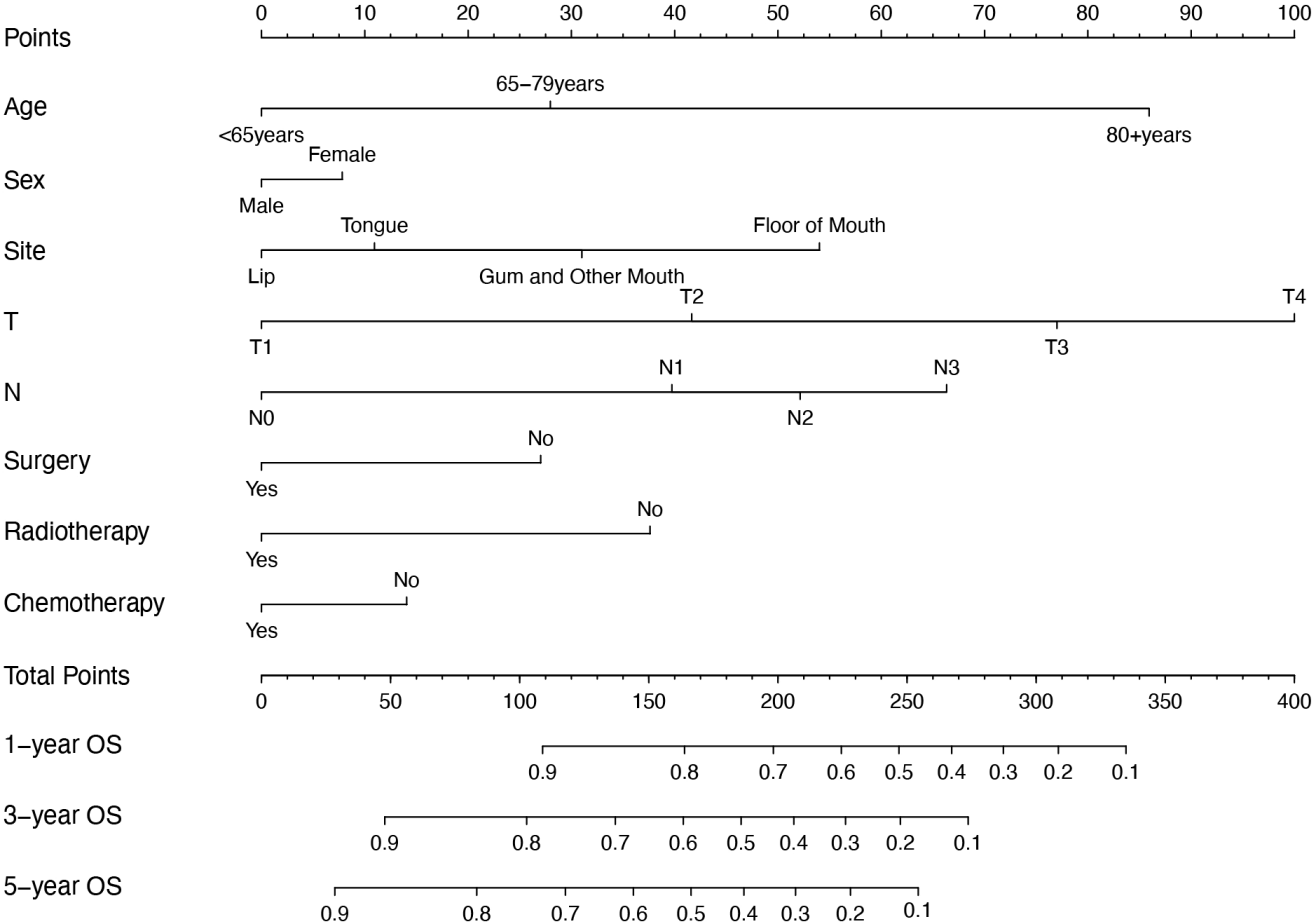
Nomogram for predicting 1-year, 3-year, and 5-year OS of OSCC patients. OS, overall survival; OSCC, oral squamous cell carcinoma.

Variables with *p* < 0.05 in the multivariate analysis were included in the model. A visual nomogram based on this model, predicting 1-, 3-, and 5-year OS, is presented in **Figure 4**. Additionally, another model was constructed that included only the TNM staging to compare its performance with that of the new model we developed.

### 3.4 Verification

In the training set, the C-index of the new model was 0.727 (95% CI: 0.724–0.730), while in the validation set, it was 0.6845 (95% CI: 0.6821–0.6869). In comparison, the TNM model exhibited a C-index of 0.6765 (95% CI: 0.673–0.680) in the training set and 0.651 (95% CI: 0.6265–0.6755) in the validation set.

**Figure 5:**
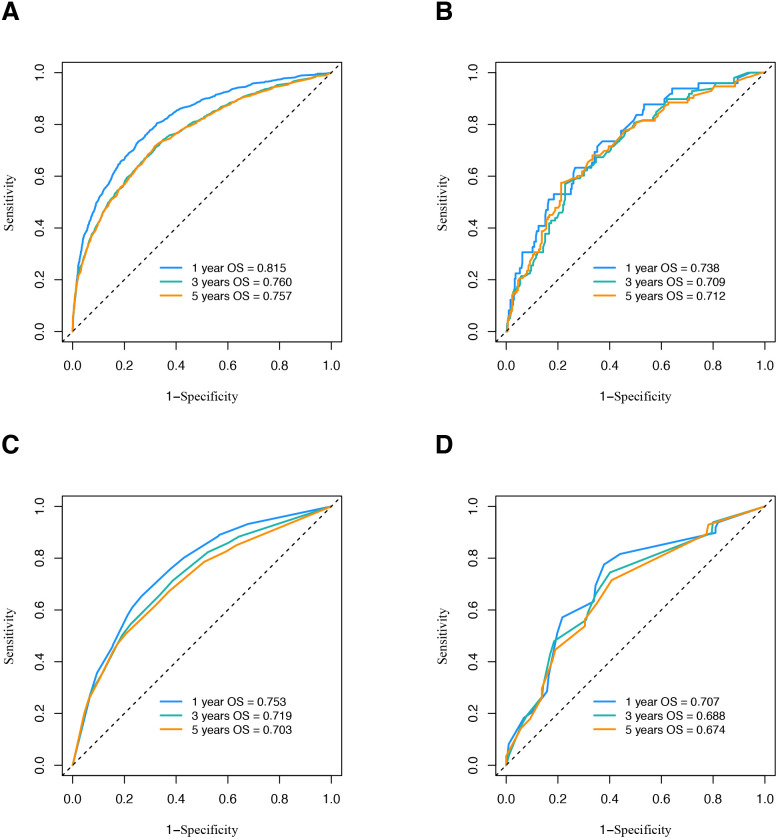
ROC curve analysis to predict OS of OSCC patients. **(A)** ROC curve of nomogram in SEER database. **(B)** ROC curve of nomogram in validation cohort. **(C)** ROC curve of traditional TNM model in SEER database. **(D)** ROC curve of traditional TNM model in validation cohort. ROC, receiver operating characteristic; OS, overall survival; OSCC, oral squamous cell carcinoma; SEER, Surveillance, Epidemiology, and End Results.

The time-dependent AUC analyses for both models across the two cohorts are presented in **Figure 5**. The new model demonstrated higher 1-, 3-, and 5-year OS AUC values compared to the TNM model, indicating superior predictive accuracy.

**Figure 6:**
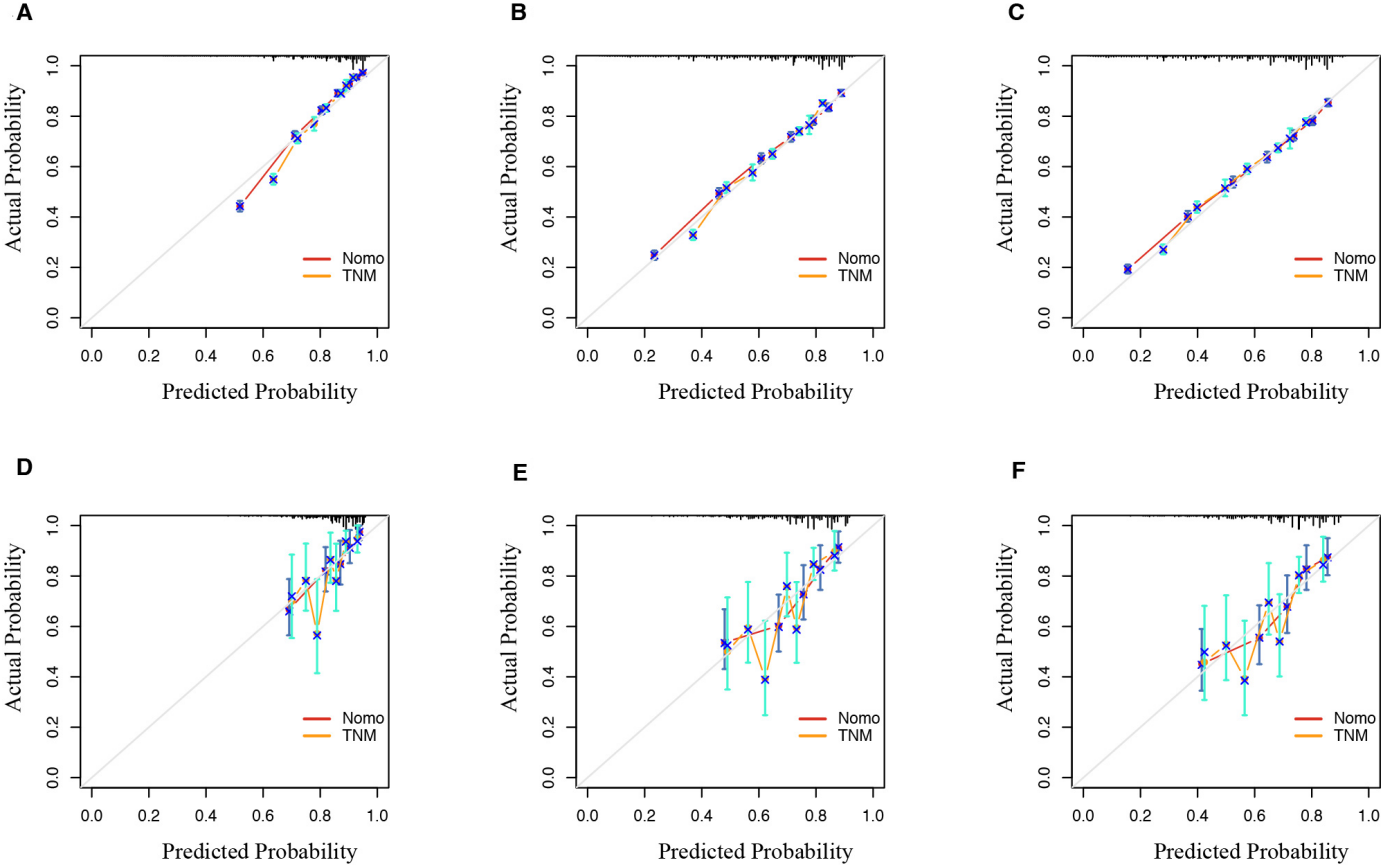
Calibration curve of the nomogram and TNM model predicted probability for 1-year, 3-year, and 5-year OS in SEER database **(A–C)** and validation set **(D–F)**. The 45° line represents an ideal match between the actual survival (y-axis) and nomogram-predicted survival (x-axis). The perpendicular line means 95% confidence intervals. OS, overall survival; SEER, Surveillance, Epidemiology, and End Results.

The calibration curves for the nomogram show high agreement between the predicted and observed probabilities of OS in both the training and validation sets (**Figure 6**).

**Figure 7:**
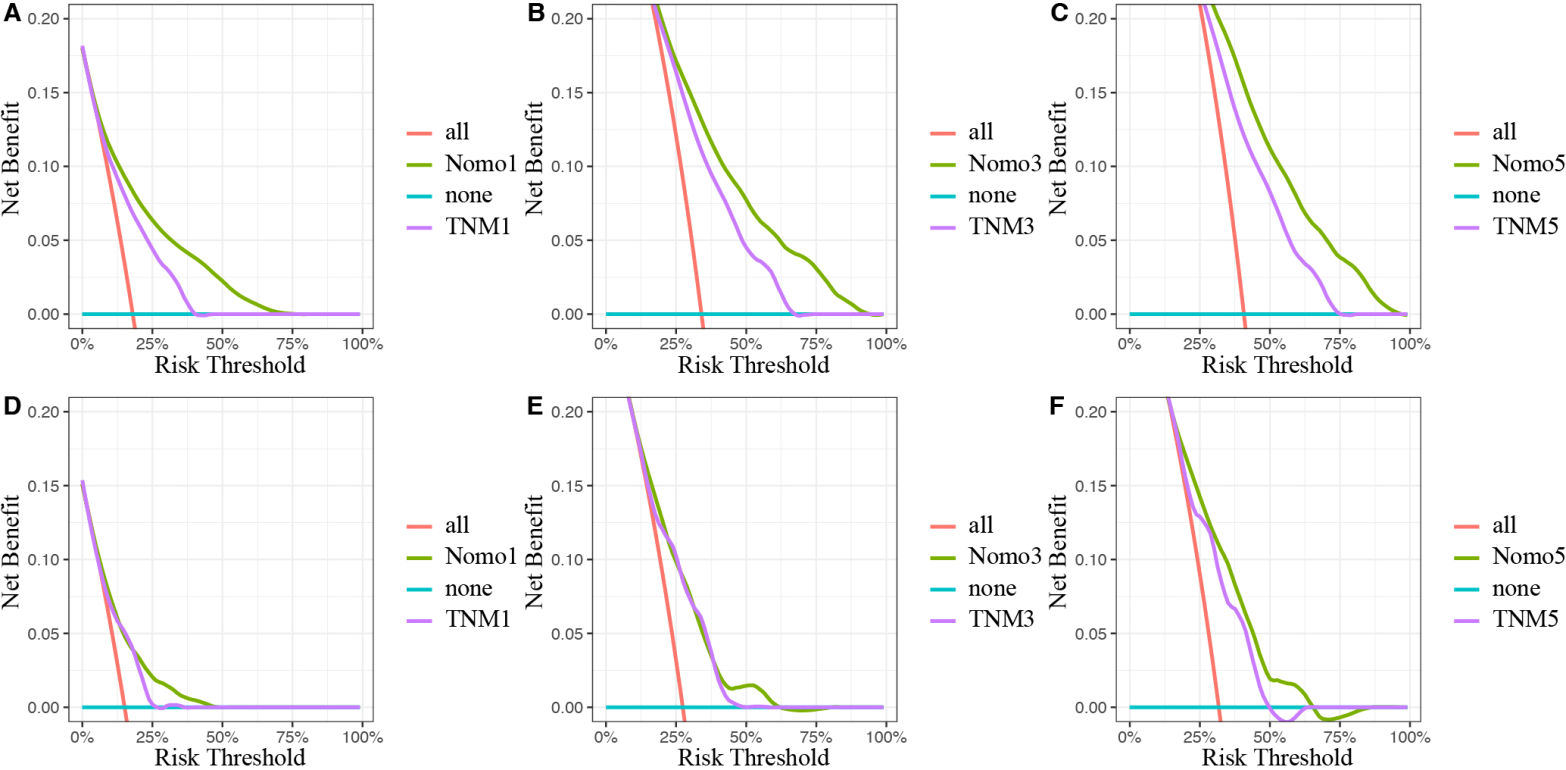
Decision curves analysis of the nomogram and traditional TNM staging model for the survival prediction of OSCC patients. **(A)** The 1-year survival benefit in SEER database. **(B)** The 3-year survival benefit in SEER database. **(C)** The 5-year survival benefit in SEER database. **(D)** The 1-year survival benefit in validation set. **(E)** The 3-year survival benefit in validation set. **(F)** The 5-year survival benefit in validation set. The x-axis indicates the threshold probabilities, and the y-axis measures the net benefit. The blue horizontal line represents the case where no patients accepted the treatment. The red line represents the case where all patients accepted the treatment. OSCC, oral squamous cell carcinoma; SEER, Surveillance, Epidemiology, and End Results.

Moreover, DCA was conducted to compare the clinical net benefit of the nomogram with that of the traditional TNM staging system. The nomogram provided a higher net benefit than the TNM staging model in both the training and validation cohorts, confirming its superior clinical utility (**Figure 7**).

### 3.5 Risk stratification

**Figure 8:**
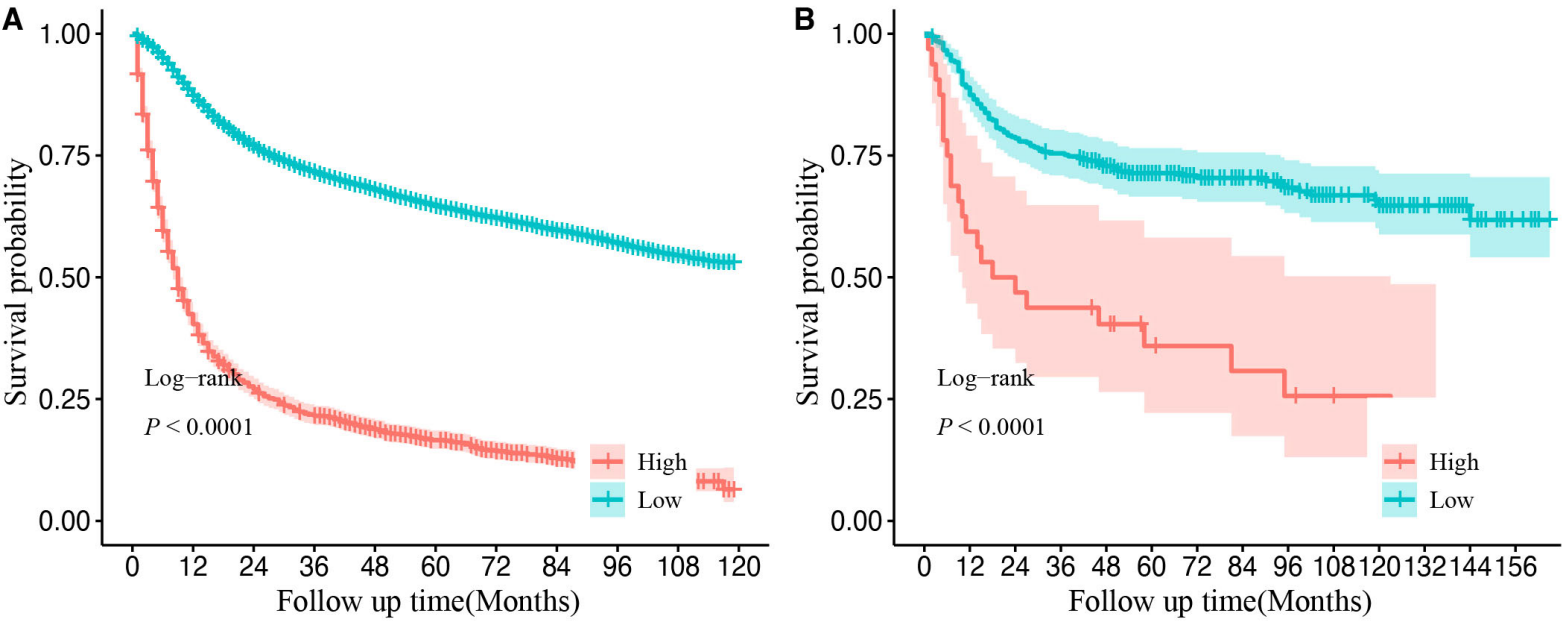
Kaplan–Meier curves of overall survival for different risk groups in the SEER database cohort **(A)** and validation cohort **(B)**. Cutoff value was obtained from the “X-tile” software. High-risk group refers to final score greater than 218 points. Low-risk group refers to final score of less than or equal to 218 points. SEER, Surveillance, Epidemiology, and End Results.

Using X-tile software, a cutoff score of 218 was identified, allowing for the effective stratification of OSCC patients into the high- and low-risk groups. The Kaplan–Meier survival curves revealed significant differences in OS between these two groups in both the SEER and CHSUMC cohorts, thereby confirming the clinical utility of the nomogram-based risk stratification for guiding patient management (**Figure 8**).

## 4 Discussion

This study developed and validated a nomogram to predict OS in patients with OSCC based on data from the SEER database and a cohort from the CHSUMC. Compared to the traditional TNM staging model, the nomogram demonstrated superior predictive performance with higher concordance indices, better calibration, and enhanced clinical utility in both the training and validation cohorts. This finding highlights the importance of incorporating additional demographic, clinical, and treatment-related factors in predictive modeling to achieve a more accurate prognosis for OSCC patients.

The baseline characteristics of the SEER and CHSUMC cohorts revealed several notable demographic and clinical differences, which could influence the interpretation and applicability of the nomogram across diverse populations. Male patients predominated in both the SEER database and the Shantou University Medical College cohort, consistent with the well-established higher incidence of OSCC among male patients compared to female patients (18). This gender disparity may reflect differential exposure to risk factors such as tobacco and alcohol use, which are known to contribute to the development of OSCC (19). The proportion of primary sites of OSCC patients was similar in the United States and China. The proportion of tongue cancer in the two cohorts was the highest, indicating that tongue cancer is the most common oral tumor, which is consistent with previous studies (2, 20, 21). In terms of staging, the SEER database showed a higher proportion of T1 tumors (38.0%) compared to CHSUMC (17.5%) and a lower proportion of T2 tumors in the SEER database (28.6%) compared to CHSUMC (44.6%) (*p* < 0.001). This suggests that patients in the CHSUMC cohort may present with more advanced disease at diagnosis, which may be caused by poor health awareness among Chinese patients (22). Additionally, the proportion of patients with the N2 stage was lower in the CHSUMC cohort (17.0% *vs.* 34.0%). This phenomenon may be related to the higher rate of detection of lymph node micrometastasis in the United States. The difference in treatment strategies adopted by OSCC patients was notable. Surgical treatment rates were significantly higher in the CHSUMC cohort, whereas SEER patients more frequently received radiotherapy or chemotherapy. This difference may be due to the higher proportion of N-positive patients in the SEER database and the fact that OSCC patients in the United States tend to choose radiotherapy and chemotherapy out of social needs and psychological, functional preservation, and other considerations (23, 24). Collectively, these baseline differences underscore the importance of considering cohort-specific factors when interpreting survival outcomes and highlight the need for model validation in diverse clinical settings to ensure broad applicability.

The study’s key observations point to significant differences in survival outcomes between patients with FOM cancer and those with tongue cancer. Patients with FOM cancer displayed a poorer prognosis, which is consistent with previous research suggesting that anatomical and biological factors contribute to the aggressive behavior of FOM tumors (25). The rich lymphatic network in the FOM facilitates early and widespread lymphatic metastasis, elevating the risk of regional and distant recurrence and, consequently, impacting OS. Additionally, patients with FOM cancer often present with advanced-stage disease due to the dormant initial symptoms associated with FOM tumors, further compounding their survival disadvantage. In contrast, tongue cancers often produce more noticeable symptoms, prompting earlier medical intervention and diagnosis, which may partly explain the improved survival rates observed in these patients. However, Farhood and colleagues studied anatomic subsite differences in oral cavity cancer mortality, and they found that FOM did not have a statistically significant difference compared to the oral tongue (26). Zelefesky et al. researched 51 FOM and oral tongue squamous cell carcinoma patients after postoperative radiotherapy and finally found that the actuarial local failure rate of oral tongue was higher than that of FOM, despite overall and disease-free survival being the same for both groups (27). Another follow-up study also observed poor local control in oral tongue squamous cell carcinoma patients, compared to other subsites (28). More studies are needed to explore the relationship between anatomical subsites and OSCC prognosis.

Another noteworthy finding of this study is the significant role of surgical intervention in improving OS among OSCC patients. Our analysis revealed that the CHSUMC cohort, which had a higher proportion of patients undergoing surgery, demonstrated a longer median OS and superior survival rates compared to the SEER cohort. This disparity can be attributed to two key factors. First, the SEER cohort included a higher proportion of N2 patients, which is associated with a worse prognosis (29). Second, the higher rate of surgical treatment in the CHSUMC cohort likely contributed to the observed survival advantage. Surgery remains widely regarded as the most effective therapy for improving prognosis in OSCC (30). A retrospective study including 934 patients by Silva PB et al. revealed that non-surgically treated OSCC patients had a lower OS than surgically treated OSCC patients (31). These results underscore the importance of surgery as a cornerstone of OSCC management and its essential role in improving survival outcomes.

Finally, a predictive model for OSCC patients containing variables including age, gender, primary site, T staging, N staging, surgery, chemotherapy, and radiotherapy was constructed. The performance of the model was better than that of the traditional TNM staging system, as evidenced by its higher C-index and ROC curve, as well as calibration curves that are closer to the ideal curve. These results indicate that the predictions generated by this model are more accurate than those based solely on the TNM staging system. Furthermore, the DCA plot reveals better performance of the model in the training set than that in the validation set. We suggest that other centers should conduct local recalibration before applying the model. Future studies should explore the performance of the model in the multicenter data to enhance its generalizability. Furthermore, compared to previous models (12–14), the nomogram developed in this study included a more comprehensive set of variables, particularly treatment-related information, which has been largely absent in earlier studies. External validation further confirmed the model’s generalizability across different populations. In terms of clinical application, this nomogram provides an invaluable tool for stratifying OSCC patients based on their risk profiles, which has direct implications for personalized treatment planning. For instance, clinicians or patients can score using the nomogram based on actual clinical information. Patients identified as high-risk through this model could be prioritized for more aggressive therapeutic strategies, such as intensified surveillance, multi-modal treatment combinations, or enrollment in clinical trials for novel therapies. This approach aligns with the contemporary trend toward personalized medicine and underscores the potential of risk stratification models in optimizing clinical decision-making. Despite the robust performance of the nomogram and its risk stratification capabilities, there are several limitations in this study. First, as a retrospective study, there will inevitably be bias due to the missing data and different treatment decisions made by different surgeons. Furthermore, other factors such as alcohol consumption, smoking, human papillomavirus (HPV) status, depth of invasion, extranodal extension, vascular or nerve invasion, specific chemotherapy regimens, and a combination of surgery, radiotherapy, and chemotherapy have been reported to influence the survival of OSCC patients (32–37). However, this information was not recorded in the SEER database. Future studies incorporating multicenter data from diverse populations and a broader range of variables could further refine and improve the model. Lastly, since this study focused on OS, future studies could explore disease-specific survival and recurrence-free survival as endpoints, which may offer further insights into the utility of this nomogram for other clinical applications.

## 5 Conclusions

In this study, we developed a novel nomogram to predict OS in patients with OSCC using data from the SEER database and validated it with an independent cohort from the CHSUMC. The nomogram demonstrated superior predictive performance compared to the traditional TNM staging system, as evidenced by higher concordance indices, better area under the ROC curves, and more accurate calibration. This nomogram offers a practical, risk-based approach for clinicians to better estimate patient outcomes and tailor treatment plans, ultimately contributing to improving patient management in OSCC.

## Data Availability

The raw data supporting the conclusions of this article will be made available by the authors, without undue reservation.

## References

[1] International Agency for Research on Cancer. Lip, oral cavity (Globocan 2020 fact sheet) (2020). Lyon: IARC. Available online at: https://gco.iarc.fr/today/data/factsheets/cancers/1-Lip-oral-cavity-fact-sheet.pdf (Accessed November 14, 2022).

[2] Siegel RL Giaquinto AN Jemal A. Cancer statistics, 2024. CA Cancer J Clin. (2024) 74:12–49. doi: 10.3322/caac.21820 38230766

[3] World Health Organization. International statistical classification of diseases and related health problems (2021). Available online at: https://icd.who.int/ (Accessed July 15, 2024).

[4] Global oral health status report: towards universal health coverage for oral health by 2030. Executive summary. Geneva: World Health Organization (2022).

[5] Shetty KSR Kurle V Greeshma P Ganga VB Murthy SP Thammaiah SK. Salvage surgery in recurrent oral squamous cell carcinoma. Front Oral Health. (2022) 2:815606. doi: 10.3389/froh.2021.815606 35156084 PMC8831824

[6] Amin MB Edge SB Greene FL. AJCC cancer staging manual. 8th ed. New York: Springer (2017).

[7] Oh LJ Asher R Veness M Smee R Goldstein D Gopalakrishna lyer N. Effect of age and gender in non-smokers with oral squamous cell carcinoma: Multi-institutional study. Oral Oncol. (2021) 116:105210. doi: 10.1016/j.oraloncology.2021.105210 33618102

[8] Suresh GM Koppad R Prakash BV Sabitha KS Dhara PS. Prognostic indicators of oral squamous cell carcinoma. Ann Maxillofac Surg. (2019) 9:364–70. doi: 10.4103/ams.ams_253_18 PMC6933976 31909017

[9] Huang X Luo Z Liang W Xie G Lang X Gou J. Survival nomogram for young breast cancer patients based on the SEER database and an external validation cohort. Ann Surg Oncol. (2022) 29:5772–81. doi: 10.1245/s10434-022-11911-8 PMC9356966 35661275

[10] Liu H Li Z Zhang Q Li Q Zhong H Wang Y. Multi-institutional development and validation of a nomogram to predict prognosis of early-onset gastric cancer patients. Front Immunol. (2022) 13:1007176. doi: 10.3389/fimmu.2022.1007176 36148218 PMC9488636

[11] Shao CY Liu XL Yao S Li ZJ Cong ZZ Luo J. Development and validation of a new clinical staging system to predict survival for esophageal squamous cell carcinoma patients: Application of the nomogram. Eur J Surg Oncol. (2021) 47:1473–80. doi: 10.1016/j.ejso.2020.12.004 33349524

[12] Wang F Zhang H Wen J Zhou J Liu Y Cheng B. Nomograms forecasting long-term overall and cancer-specific survival of patients with oral squamous cell carcinoma. Cancer Med. (2018) 7:943–52. doi: 10.1002/cam4.1216 PMC5911576 29512294

[13] Zhang XY Xie S Wang DC Shan XF Cai ZG. Prognosis and nomogram prediction for patients with oral squamous cell carcinoma: A cohort study. Diagnostics (Basel). (2023) 13:1768. doi: 10.3390/diagnostics13101768 37238252 PMC10217586

[14] Wang W Zhang Q Thomson P Sharma D Ramamurthy P Choi SW. Predicting oral cancer survival-Development and validation of an Asia-Pacific nomogram. J Oral Pathol Med. (2023) 52:628–36. doi: 10.1111/jop.13454 37247328

[15] National Cancer Institude. Surveillance, Epidemiology, and End Results Program. Available online at: http://seer.cancer.gov (Accessed July 15, 2024).

[16] Camp RL Dolled-Filhart M Rimm DL. X-tile: a new bio-informatics tool for biomarker assessment and outcome-based cut-point optimization. Clin Cancer Res. (2004) 10(21):7252–9. doi: 10.1158/1078-0432.CCR-04-0713 15534099

[17] Stensrud MJ Hernán MA. Why test for proportional hazards? JAMA. (2020) 323:1401–2. doi: 10.1001/jama.2020.1267 PMC11983487 32167523

[18] Conway DI Stockton DL Warnakulasuriya KA Ogden G Macpherson LM. Incidence of oral and oropharyngeal cancer in United Kingdom (1990-1999) -- recent trends and regional variation. Oral Oncol. (2006) 42(6):586–92. doi: 10.1016/j.oraloncology.2005.10.018 16469526

[19] Conway DI Stockton DL Warnakulasuriya KA Ogden G Macpherson LM. Interaction between tobacco and alcohol use and the risk of head and neck cancer: pooled analysis in the International Head and Neck Cancer Epidemiology Consortium. Cancer Epidemiol Biomarkers Prev. (2009) 18:541–50. doi: 10.1158/1055-9965.EPI-08-0347 PMC3051410 19190158

[20] Siegel RL Miller KD Jemal A. Cancer statistics, 2020. CA Cancer J Clin. (2020) 70:7–30. doi: 10.3322/caac.21590 31912902

[21] Zanoni DK Montero PH Migliacci JC Shah JP Wong RJ Ganly I. Survival outcomes after treatment of cancer of the oral cavity (1985-2015). Oral Oncol. (2019) 90:115–21. doi: 10.1016/j.oraloncology.2019.02.001 PMC6417804 30846169

[22] Zhou XH Huang Y Yuan C Zheng SG Zhang JG Lv XM. A survey of the awareness and knowledge of oral cancer among residents in Beijing. BMC Oral Health. (2022) 22:367. doi: 10.1186/s12903-022-02398-6 36031600 PMC9420274

[23] Lewis CM Ajmani GS Kyrillos A Chamberlain P Wang CH Nocon CC. Racial disparities in the choice of definitive treatment for squamous cell carcinoma of the oral cavity. Head Neck. (2018) 40:2372–82. doi: 10.1002/hed.25341 PMC6563920 29947066

[24] Wang CP Liao LJ Chiang CJ Hsu WL Kang CJ Wang CC. Patients with oral cancer do not undergo surgery as primary treatment: A population-based study in Taiwan. J Formos Med Assoc. (2020) 119:392–8. doi: 10.1016/j.jfma.2019.06.011 31280909

[25] Lo WL Kao SY Chi LY Wong YK Chang RC. Outcomes of oral squamous cell carcinoma in Taiwan after surgical therapy: factors affecting survival. J Oral Maxillofac surgery: Off J Am Assoc Oral Maxillofac Surgeons. (2003) 61:751–8. doi: 10.1016/s0278-2391(03)00149-6 12856245

[26] Farhood Z Simpson M Ward GM Walker RJ Osazuwa-Peters N. Does anatomic subsite influence oral cavity cancer mortality? A SEER database analysis. Laryngoscope. (2019) 129:1400–6. doi: 10.1002/lary.27490 30408182

[27] Zelefsky MJ Harrison LB Fass DE Armstrong J Spiro RH Shah JP. Postoperative radiotherapy for oral cavity cancers: impact of anatomic subsite on treatment outcome. Head Neck. (1990) 12:470–5. doi: 10.1002/hed.2880120604 2258285

[28] Zelefsky MJ Harrison LB Fass DE Armstrong JG Shah JP Strong EW. Postoperative radiation therapy for squamous cell carcinomas of the oral cavity and oropharynx: impact of therapy on patients with positive surgical margins. Int J Radiat Oncol Biol Phys. (1993) 25:17–21. doi: 10.1016/0360-3016(93)90139-m 8416876

[29] Sim YC Hwang JH Ahn KM. Overall and disease-specific survival outcomes following primary surgery for oral squamous cell carcinoma: analysis of consecutive 67 patients. J Korean Assoc Oral Maxillofac Surg. (2019) 45:83–90. doi: 10.5125/jkaoms.2019.45.2.83 31106136 PMC6502750

[30] Shaw RJ O’Connell JE Bajwa M. Basic surgical principles and techniques. In: Warnakulasuriya S Greenspan J, editors. Textbook of Oral Cancer. Springer, Cham (2020). doi: 10.1007/978-3-030-32316-5_20

[31] Silva PB Lemos JV Borges MM do Rêgo TJ Dantas TS Leite CH. Prognostic factors on surgically and non-surgically treated oral squamous cell carcinoma: Advances in survival in fifteen years of follow up. J Clin Exp Dent. (2021) 13:e240–9. doi: 10.4317/jced.57477 PMC7920565 33680326

[32] Lee SU Moon SH Choi SW Cho KH Park JY Jung YS. Prognostic significance of smoking and alcohol history in young age oral cavity cancer. Oral Dis. (2020) 26:1440–8. doi: 10.1111/odi.13432 32430951

[33] Andersen AO Jensen JS Jakobsen KK Stampe H Nielsen KJ Wessel I. The impact of tobacco smoking on survival of patients with oral squamous cell carcinoma: a population-based retrospective study. Acta Oncol. (2022) 61:449–58. doi: 10.1080/0284186X.2022.2033830 35114883

[34] Christianto S Li KY Huang TH Su YX. The prognostic value of human papilloma virus infection in oral cavity squamous cell carcinoma: A meta-analysis. Laryngoscope. (2022) 132:1760–70. doi: 10.1002/lary.29996 34953144

[35] Wunschel M Neumeier M Utpatel K Reichert TE Ettl T Spanier G. Staging more important than grading? Evaluation of Malignancy grading, depth of invasion, and resection margins in oral squamous cell carcinoma. Clin Oral Investig. (2021) 25:1169–82. doi: 10.1007/s00784-020-03421-2 PMC7878266 32601998

[36] Arun I Maity N Hameed S Jain PV Manikantan K Sharan R. Lymph node characteristics and their prognostic significance in oral squamous cell carcinoma. Head Neck. (2021) 43:520–33. doi: 10.1002/hed.26499 33021340

[37] Spoerl S Gerken M Fischer R Mamilos A Spoerl S Wolf S. Lymphatic and vascular invasion in oral squamous cell carcinoma: Implications for recurrence and survival in a population-based cohort study. Oral Oncol. (2020) 111:105009. doi: 10.1016/j.oraloncology.2020.105009 33032181

